# N400 Indexing the Motion Concept Shared by Music and Words

**DOI:** 10.3389/fpsyg.2022.888226

**Published:** 2022-06-17

**Authors:** Tongquan Zhou, Yulu Li, Honglei Liu, Siruo Zhou, Tao Wang

**Affiliations:** ^1^School of Foreign Languages, Southeast University, Nanjing, China; ^2^College of Chinese Language and Literature, Qufu Normal University, Qufu, China; ^3^School of Music, Qufu Normal University, Rizhao, China; ^4^Department of Chinese Language and Literature, Yonsei University, Seoul, South Korea; ^5^School of Psychology, Qufu Normal University, Qufu, China

**Keywords:** verbs, nouns, music, motion concept, embodiment

## Abstract

The two event-related potentials (ERP) studies investigated how verbs and nouns were processed in different music priming conditions in order to reveal whether the motion concept *via* embodiment can be stimulated and evoked across categories. Study 1 (Tasks 1 and 2) tested the processing of verbs (action verbs vs. state verbs) primed by two music types, with tempo changes (accelerating music vs. decelerating music) and without tempo changes (fast music vs. slow music) while Study 2 (Tasks 3 and 4) tested the processing of nouns (animate nouns vs. inanimate nouns) in the same priming condition as adopted in Study 1. During the experiments, participants were required to hear a piece of music prior to judging whether an ensuing word (verb or noun) is semantically congruent with the motion concept conveyed by the music. The results show that in the priming condition of music with tempo changes, state verbs and inanimate nouns elicited larger N400 amplitudes than action verbs and animate nouns, respectively in the anterior regions and anterior to central regions, whereas in the priming condition of music without tempo changes, action verbs elicited larger N400 amplitudes than state verbs and the two categories of nouns revealed no N400 difference, unexpectedly. The interactions between music and words were significant only in Tasks 1, 2, and 3. Taken together, the results demonstrate that firstly, music with tempo changes and music without tempo prime verbs and nouns in different fashions; secondly, action verbs and animate nouns are easier to process than state verbs and inanimate nouns when primed by music with tempo changes due to the shared motion concept across categories; thirdly, bodily experience differentiates between music and words in coding (encoding and decoding) fashion but the motion concept conveyed by the two categories can be subtly extracted on the metaphorical basis, as indicated in the N400 component. Our studies reveal that music tempos can prime different word classes, favoring the notion that embodied motion concept exists across domains and adding evidence to the hypothesis that music and language share the neural mechanism of meaning processing.

## Introduction

Music and language as the two important communicating systems for human beings are comparable in multiple dimensions (e.g., acoustic features, emotions, and meanings) but mainly in two aspects, syntax and semantics. Syntactically, music and language have similar hierarchical configurations whereby discrete structural elements are combined into sequences (Patel, 2008, p. 241). As an illustration, a section in music is composed of motifs and phrases while a sentence in a language is composed of words and phrases in a hierarchical fashion. This syntactic comparability has been demonstrated in a couple of neuropsychological and neuroimaging studies (e.g., Jentschke et al., 2014; Chiang et al., 2018). Semantically, both music and language are adopted to convey the information that can be interpreted and comprehended by others, despite the point that “the meaning evoked by music is far less specific than meaning evoked by language” (Slevc and Patel, 2011). In Koelsch et al.'s (2004) event-related potential (ERP) study using different types of contexts to prime the processing of words, N400 was elicited by nouns when preceded by either semantically unrelated musical excerpts or semantically unrelated sentences. Afterward, numerous researches converge to reveal the psychological reality of musical meaning similar to linguistic meaning as indexed by N400, in both music-priming-words conditions and words/ sentences-priming-music conditions (e.g., Steinbeis and Koelsch, 2008; Daltrozzo and Schön, 2009a,b; Koelsch, 2011). So to speak, meanings are encoded diversely by music and language, but their semantics does partly overlap at least from the perspective of meaning processing.

Musical meaning is abundant in kinds. According to Koelsch (2011), musical meaning can arise from extra-musical sign qualities, intra-musical structural relations, musicogenic effects, the establishment of a unified coherent sense out of “lower-level” units, or musical discourse, which together are generalized into three fundamentally different classes of meaning, extra-musical meaning, intra-musical meaning, and musicogenic meaning. Related to our study is the extra-musical meaning^1^ which “emerges from the interpretation of musical information with reference to the extra-musical world”, specified as three dimensions—iconic musical meaning, indexical musical meaning, and symbolic musical meaning (Koelsch, 2011). The meaning of musical motion (i.e., motion concept) pertains to iconic musical meaning used to imitate the sounds and qualities of objects or qualities of abstract concepts, in accordance with Eitan and Granot (2006) that music is able to evoke a sense of motion in a listener and with Patel (2008, p. 331) that music can evoke semantic concepts. In light of cognitive linguistics, our understanding of musical motion is completely metaphoric, grounded by our bodily experiences of physical motion (Johnson and Larson, 2003). That is, the motion concept in music can be metaphorically understood *via* embodiment, in fact, a cross-domain mapping from physical motion to musical motion involving our participation (Sloboda, 1998; Todd, 1999; Larson, 2002, 2004; Johnson and Larson, 2003; Eitan and Granot, 2006; Hedger et al., 2013). On the basis of embodiment, the motion concept is encoded not only by music but also by words in the language, for words are the basic categories used to represent an entity, actions, or their relevant features (Wolter et al., 2015). Verbs and nouns as the two major word classes in language are used to represent dynamic objects and static objects separately in general (Shao and Liu, 2001), yet nouns can communicate dynamic characteristics in some ways (as illustrated below). As a result, it is possible to use music to prime verbs and nouns on the shared motion concept basis.

In Mandarin Chinese, verbs have two types of meaning, static meaning and dynamic meaning (Dai et al., 1995), respectively related to state verbs and action verbs. Based on our bodily experience, action verbs and state verbs represent two different subcategories in terms of the motion features on their own. As a consequence, state verbs are often perceived to encode physical state or property and are hence called low-motion verbs, while action verbs, often called high-motion verbs, embrace more motion information than state verbs (Muraki et al., 2020). This point of view is basically consistent with Grossman et al.'s (2002) found that motion-related verbs involve more sensorimotor experience than state verbs, accordingly yielding the relatively easier processing of action verbs. For nouns, animacy is a good indicator for judging their related motion information. According to Weckerly and Kutas (1999), animate nouns as ideal actors in sentences have a strong possibility to perform actions and consequently contain more motion information compared to inanimate nouns. Experiments show that animacy can be clearly distinguished by infants <1 year old based on the motion clues with dynamic or static information (Pauen and Träuble, 2009; Träuble et al., 2014). All these studies suggest that verbs and nouns can convey motion concept but the concept is differently encoded not only between the two-word classes but also between their subcategories.

Motion concept is differently mapped onto music and words. In music, tempo as an expression of extra-musical meaning is used to convey the motion concept metaphorically (Todd, 1999; Johnson and Larson, 2003; Eitan and Granot, 2006; Zhou et al., 2015), involving physical motion and motion imagery. For instance, music tempos with acceleration and deceleration can elicit images of increasingly and decreasingly speeded motion, respectively (Eitan and Granot, 2006; Savaki and Raos, 2019). In the view of Hedger et al. (2013), relative to statistically fast and slow music, accelerating music and decelerating music can better prime pictures in motion and at rest, respectively. This suggests that the music with tempo changes (accelerating music, decelerating music) can better convey motion concept than music without tempo changes (statistically fast and slow music), for the tempo changes in a single piece with acceleration or deceleration may be more apparent and expectable (Hedger et al., 2013). Different from the explicit mapping of motion concept onto music tempo, the motion concept is encoded by verbs and nouns in language implicitly. That is, one's speaking of a verb or a noun evokes his motion concept covertly and subconsciously. Grounded by this difference, using music tempo to prime verbs and nouns appears more salient than the other way around.

The motivation of music and words able to convey motion concept is well-explained by the embodiment theories and the theory of embodiment semantics. As one of the classical embodiment theories, the perceptual symbols hypothesis (Barsalou, 1999) holds that the symbols are assumed to be the residues of a perceptual experience stored as patterns in the brain for activation. In light of the hypothesis, motion experience is to be activated and simulated as individuals process the motion concept shared by music and words. In music, the acoustic properties of music tempos mimic the properties of physical motion. Similar to the perception of music, the motion concept, which is abstract and implicit in language, is metaphorically encoded by lexical meaning (Hauk et al., 2004; Wolter et al., 2015). To illustrate it, the word –奔跑 “*benpao/*run” is first understood semantically and then its motion attributes as one of its lexical meanings was decoded metaphorically. Also, the motion concept of words is related to embodiment, our sensory-motor experiences (Barsalou, 1999). Likewise, the theory of embodiment semantics claims that the comprehension of lexical meaning is based on our bodily experience and can activate the brain regions responsible for perception, emotion, and action (de Vega et al., 2008; Horchak et al., 2014). This claim has been justified in an fMRI study that sensorimotor experiential traces are activated while processing words referring to an action, e.g., “kicking” can activate the premotor cortex in the brain as actual kicking movement being performed (Hauk et al., 2004).

As stated above, N400 turned out to be an index of semantic incongruity related to extramusical meaning and linguistic meaning. Yet to date, the literature using music to prime words' meaning or motion concept has been confined to three studies by Koelsch et al. (2004), Hedger et al. (2013), and Zhou et al. (2015). Hedger et al. (2013) conducted a behavioral study to reveal that accelerating music and decelerating music can better prime motion concept than fast music and slow music and the incongruency motion relations between tempos and pictures cost more time than the congruency. In Koelsch et al. (2004)'s ERP study, music excerpts were proved to be as valid as sentences to facilitate semantically congruent words, as revealed by the N400 in both music and sentence conditions. Similarly, Zhou et al. (2015) drew up music excerpts to prime semantically congruent and incongruent pictures in a set of ERP experiments to indicate that incongruent pairs elicited a larger N400 than the congruent pairs over the anterior and central regions, further justifying the role of N400 in revealing the motion concept conveyed by music. The three studies converge to show that music can convey motion concept and other meanings related to words or pictures. Nevertheless, scrutiny of their experiments uncovers some gaps to be filled: for one thing, word types as the target stimuli were not rigidly manipulated–e.g., concrete nouns were confused with abstract nouns, and mono-category words confused with multi-category words in Koelsch et al. (2004) and the heterogeneity of stimuli may fail to reveal the priming effect as anticipated; for the other, the motion concept-based (in)congruency was established between music and pictures as in Hedger et al. (2013) and Zhou et al. (2015), leaving an open question whether music can exert influence on verbal stimuli like verbs and nouns so that motion concept as a putative shared meaning can be evoked across more domains.

Against the above background, the current study referring to Hedger et al. (2013) and Zhou et al. (2015) utilizes two ERP experiments (Studies 1 and 2) to explore (1) whether music tempos can prime Chinese verbs and nouns, (2) how the congruency between music and words is established on their shared motion concept basis, and (3) how the four classes of verbs and nouns are distinguished from the perspective of processing under music priming conditions. Specifically, Study 1 tested how the two types of music (with vs. without tempo changes) affect the processing of two sub-classes of verbs (action verbs vs. state verbs) while Study 2 tested how the same two types of music affected the processing of two sub-classes of nouns (animate nouns vs. inanimate nouns). In association with previous relevant research, we make the predictions as below:

First, music with tempo changes could better facilitate the processing of verbs and nouns than music without tempo changes, yielding a reduced N400 effect by the words;

Second, on the basis of the shared motion concept, the incongruent pairs relative to the congruent pairs between music and words may elicit larger N400 amplitudes in the words;

Third, action verbs and animate nouns should be easier to process than state verbs and inanimate nouns, respectively. This relative ease was due to individuals' sensorimotor experience with more motion information related to the two subclasses of words.

## Study 1

As a rule, verbs are used to typically represent actions, and music is characterized by tempo changes, a way of motion representation. Based on this conceptual similarity, the first study investigated whether and how music could activate the processing of verbs by virtue of the cross-modality concept priming paradigm. Two types of music (with or without tempo changes) were selected to prime two types of Chinese verbs (action verbs or state verbs). The whole experiment was composed of two tasks by a within-subjects design, in which Task 1 adopted the music with tempo changes (accelerating music vs. decelerating music) and Task 2 with the music without tempo changes (fast music vs. slow music) to prime verbs (action verbs vs. state verbs).

**Figure 1:**
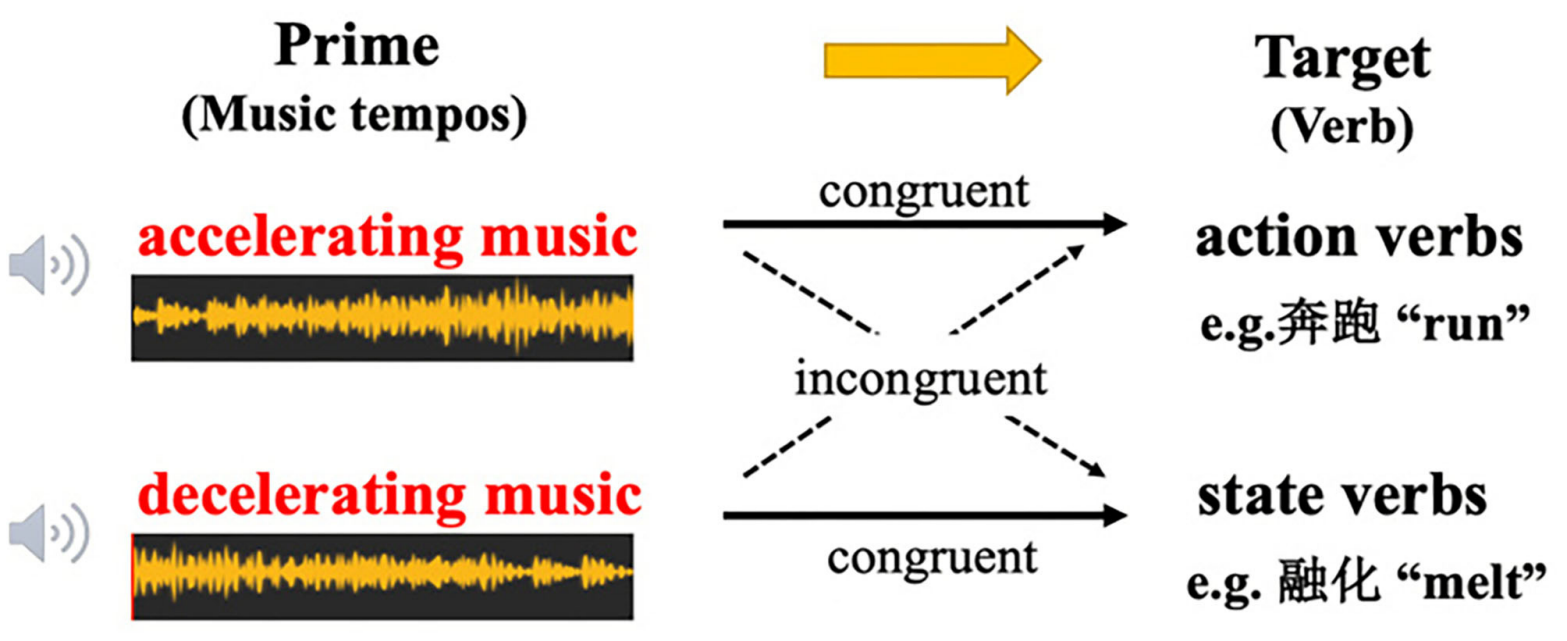
Design of the cross-modality priming paradigm in task 1 of Experiment 1. Music tempos were used as primes, and verbs were used as targets.

In light of the above-mentioned music type and verb type, four pairs of priming conditions were designed for Task 1 as shown in Figure 1: accelerating music—action verb pair, decelerating music—state verb pair to constitute a congruent relation whereas decelerating music—action verb pair and accelerating music—state verb pair to constitute an incongruent relation. Task 2 was similar to Task 1, with the only difference that the prime stimuli were music without tempo changes (fast music or slow music). Specifically, fast music—action verb pair and slow music—state verb pair constituted a congruent relation whereas slow music—action verb pair and fast—state verb pair constituted an incongruent relation. Such a design was referred to Hedger et al.'s (2013) finding that accelerating music and decelerating music could better prime pictures in motion and at rest separately, and in our experiment action verbs and state verbs were adopted to replace the pictures in motion and at rest so as to build up congruent and incongruent stimulus pairs.

### Methods

#### Participants

A total of 40 Qufu Normal University students (age: *M* = 20.1 years, *SD* = 1.5 years, ranging from 18 to 24 years of age; gender: 36 women, four men) were recruited to participate in the experiment as paid volunteers. All the participants are native Chinese speakers, right-handed in terms of the Edinburgh Handedness Inventory (Oldfield, 1971), with normal hearing and normal or corrected to normal vision and no history of psychiatric or neurological diseases. They have no musical experience (none had received professional musical training or played any instruments). All participants signed a formal written consent before the experiment. The experiment was approved by the Ethics Committee of Qufu Normal University. The data of two participants were discarded due to excessive drift artifacts during the experiment. Therefore, the data to enter into *post-hoc* analysis consisted of 38 participants (age: *M* = 20.03 years, *SD* = 1.46 years; gender: 34 women, four men).

### Stimuli Construction

Priming stimuli were music motifs. In Task 1, 20 music motifs were created as the priming stimuli, which were subdivided into 10 accelerating music motifs and ten decelerating music motifs and reduplicated twice for each. In Task 2, the priming music was changed into the one without tempo-change motifs (fast music and slow music). All the music motifs were created by a MIDI controller and audio editor 3.2.9, 44 kHz, 16-bit resolution, with an average duration of 10 s. These music stimuli consisted of two oscillating notes which were alternatively processed to form a rhythm (see Hedger et al., 2013 for similar manipulations). Participants listened to the music *via* Sony MRD-XB55AP headphones prior to making (in) congruency judgment between the music and the visualized words on the computer screen. In Task 1, accelerating music (which began with a tempo of 120 BPM and ended with 600 BPM) represented the music with strong motion information while decelerating music (from 600 to 120 BPM) represented the music with static motion information. In Task 2, fast music motifs (at 600 BPM from the beginning of a note to its end) and slow music motifs (at 120 BPM) are linked with music with strong motion and static motion information, respectively. Additionally, 20 music motifs with irregular tempo changes were selected as fillers for each task.

The target stimuli were Chinese verbs, comprised of 20 action verbs and 20 state verbs in each task, yielding 40 target stimuli for each task. No verbs in Task 1 re-occurred in Task 2. Target words were taken from the CCL corpus (Center for Chinese Linguistics, Peking University) with high frequency. According to Hu et al. (1989), action verbs were selected by referring to the following criteria: (1) the verbs signal action other than state; (2) the action is autonomous; (3) the action is concrete other than virtual or abstract; (4) each action doer (agent) has the strong executive ability and individual motivation. In accordance with the definition of state verb by different scholars (Yuan, 1998; Chen, 2002; Ma, 2005), forty state verbs were selected by referring to the following criteria: (1) the verbs are non-autonomous; (2) the verbs signal sustainable state or property other than action; (3) the verbs are not used perfectively; (4) verbs are non-bodily and have no spatial displacement. The stoke number of words were balanced (action verb: *M* = 17.3, *SD* = 4.04; state verb: *M* = 18.7, *SD* = 4.071), with independent *t*-test showing that they were not systematically different [*t*_(78)_ = −1.544, *p* = 0.127]. A total of 20 conjunctions were selected as fillers for each task.

#### Normalization of Materials

In order to obtain the stimuli for experiments, we normalized the materials by testing the motion attributes and imaginability of verbs and the relatedness between music and verbs prior to the experiment.

In the motion attributes test and imaginability test, action verbs and state verbs were selected to represent high-motion words and low-motion words, respectively, and all verbs with high imaginability. Before the experiment, 60 action verbs and 60 state verbs on a seven-point scale were tested by 100 participants who did not participate in the formal experiment, ranging from −3 (very low in motion) to +3 (very high in motion) the test motion attribute and −3 (very low imaginability) to +3 (very high imaginability) for the test of imaginability. Finally, 40 action verbs and 40 state verbs were selected as experimental materials. The average score of action verbs with high motion information evaluation was 2.165 (*SD* = 0.213) and state verbs with low motion information evaluation was −2.105 (*SD* = 0.214). In terms of imaginability, the average scores of action verbs and state verbs were 2.045 (*SD* = 0.281) and 2.016 (*SD* = 0.199). An independent samples *t*-test showed that the two classes of verbs were significantly different from each other with regard to motion information [*t*_(78)_ = 89.483, *p* < 0.01] but not significantly different in imaginability [*t*_(78)_ = 0.523, *p* = 0.603].

In order to examine whether music is related to verbs with respect to the motion concept, we tested their congruency relations on the basis of a seven-point scale, from −3 (strong unrelated) to +3 (strong related), with 0 signaling uncertainty. Only the excerpts with a mean rating score of higher than +1 or lower than −1 were selected as experimental materials. Results showed that the mean scores of the congruent groups in Tasks 1 and 2 were 2.011 (*SD* = 0.246) and 1.876 (*SD* = 0.246), respectively, and the mean rating scores of the incongruent groups in Tasks 1 and 2 were −1.966 (*SD* = 0.271) and −1.734 (*SD* = 0.182), respectively, suggesting that people can well-establish the congruency between music and nouns perceptively. Besides, *t*-test revealed that the congruent pairs and the incongruent pairs were significantly different in both Task 1 [*t*_(38)_ = 48.664, *p* < 0.01] Task 2 [*t*_(38)_ = 52.819, *p* < 0.01]. According to the rating results, 40 pairs in each task (10 pairs in each condition) were chosen as final experiment materials for subsequent experiments. Items and conditions were counterbalanced in each task. All the materials were presented in random order and designed *via* E-prime 3.0 (Psychology Software Tools, Inc.).

#### Procedures

**Figure 2:**
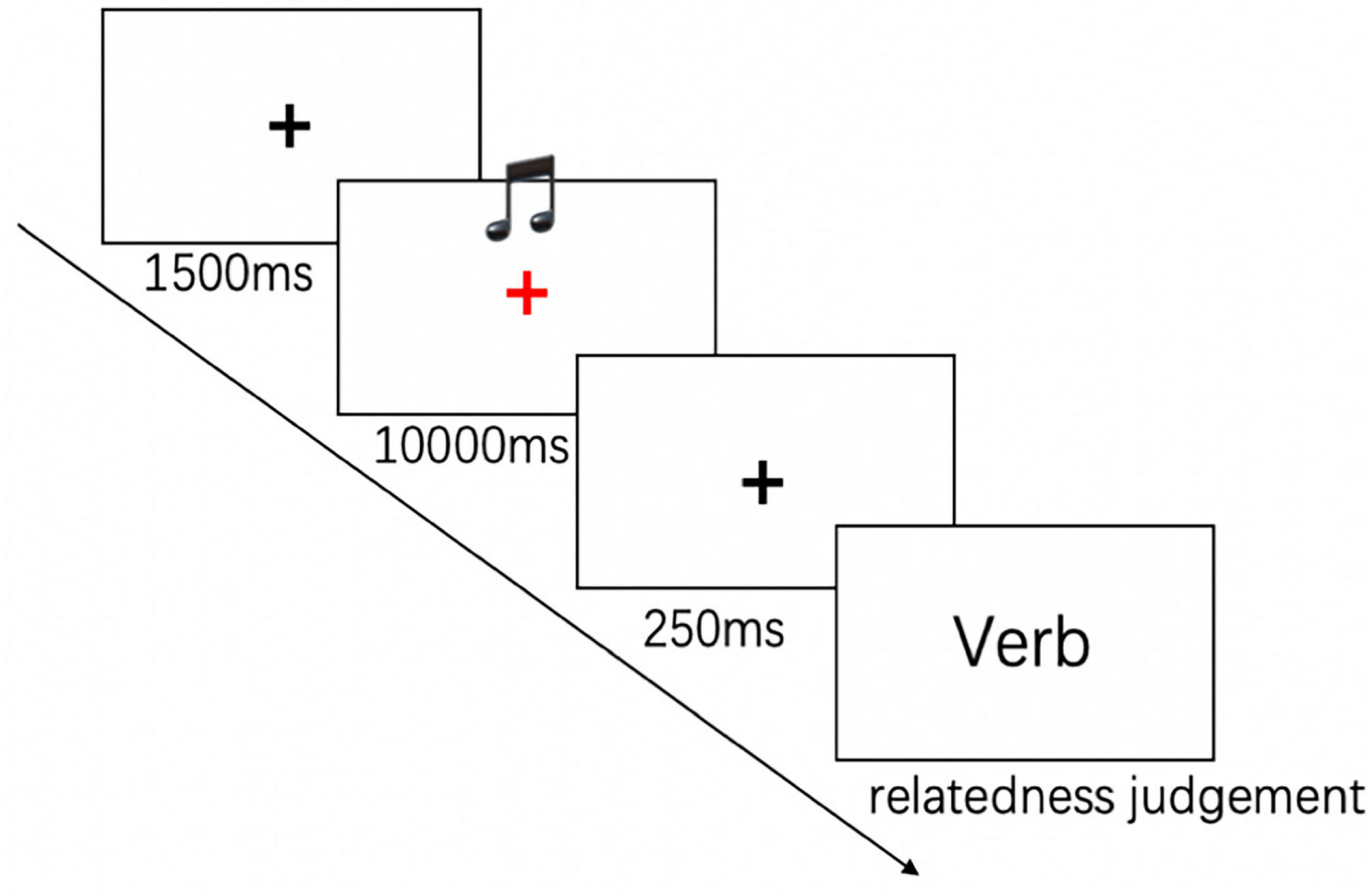
The procedure of Experiment 1.

Participants were seated on a comfortable chair in front of the computer screen approximately one meter away in a soundproof room. In each trial, the participants heard a piece of music *via* the earphone they were wearing, followed by a verb at the center of the screen on a computer. Their tasks were to as quickly as possible judge whether or not the verb was congruent with the music motif in terms of motion information (pressing the button “F” for congruent or “J” for incongruent), with the procedure as shown in Figure 2. As the experiment started, there appeared a black fixation cross (lasting 1,500 ms) at the center of the screen against a white background. As the cross turned red, the music as the priming stimulus was broadcasted for 10 s. Afterward, a second black fixation cross came out as an interval for 250 ms until each participant made the congruency judgment on the target verb. No response time limitation was set for the judgment on the verb. All participants were required not to blink their eyes as much as possible with the exception of the interval (1,500 ms black fixation cross) between each trail. The order of all the trails was presented randomly, with each trail for once. The entire experiment lasted ~14 min.

#### EEG Recording and Preprocessing

The EEG data were recorded by AC amplifiers from 64 scalp locations of the International 10–20 system. The electrodes placed on the left and right mastoids served as the reference, and the electrode between Fz and Cz was selected as the ground. To filter the eye movement and eye blink, the data of the horizontal electrodes placed on the canthus of each eye and vertical electrodes above and below the left eye were recorded. Impedances of the electrodes were kept below 5 kΩ. EEG data were digitized with the rate of 1,000 Hz and amplified and filtered within a band-pass of 0.1 and 30 Hz. Trails with artifacts (eye movements, head movements) exceeding the amplitude of ±200 μV at any channel were excluded. Raw EEG data were preprocessed by NeuroScan SynAmps2 8050 (Compumedics Neuroscan) and Curry 8 software (Compumedics Neuroscan). Further processing was carried out by EEGLAB 14.1.1 (Delorme and Makeig, 2004) in MATLAB 2013b (MathWorks).

#### ERP Data Analysis

All the data were analyzed by computing the mean amplitudes under each condition for each task. ERP data were segmented from 200 ms before to 800 ms after the onset of the target, with a 200 ms pre-stimulus correct baseline. Based on the previous studies on the N400, a time window from 300 to 500 ms after target-stimulus onset was chosen for statistical analysis.

Preliminary analysis of congruency level did not show the processing advantages of congruent pairs or incongruent pairs, suggesting the relations between music and words are more complex. Given that different verbs have different attributes and processing patterns (e.g., Amsel and Cree, 2013; Muraki et al., 2020: nonbodily state abstract verbs can elicit a larger N400 component than concrete verbs), we referred to Hedger et al. (2013) to reanalyze the data by selecting the music tempos and verbs as conditions so as to observe the processing of each type of word. The result shows that the motion concept indexed by N400 can be revealed by different types of music and words but not by their congruency levels.

To test the distribution of the effects, nine-item regions of interest (ROIs) were selected, with details shown in Table 1. For statistical analysis, ERPs were analyzed by repeated-measures ANOVAs to test the effects among music, verb, hemisphere, and regions. Nine regions of interest were divided into lateral electrode regions and midline electrode regions. The mean amplitudes of the lateral and midline electrode regions were computed separately for analysis. For the lateral electrodes, music (accelerating music and decelerating music in Task 1, fast music and slow music in Task 2), verb (action verbs and state verbs), region (anterior, central, and posterior) and hemisphere (left hemisphere and right hemisphere) served as within-subjects factors. For the midline electrodes, music (accelerating music and decelerating music in Task 1, fast music and slow music in Task 2), verb (action verbs and state verbs), and regions (anterior, central, and posterior) served as within-item factors.

**Table 1:** Electrode channels in each region of interest (ROI).

**ROI**	**Left**	**Central**	**Right**
Anterior	FP1, AF3, F1, F3, F5, F7	FPz, Fz	FP2, AF4, F2, F4, F6, F8
Central	FC3, FC5, C3, C5, CP3, CP5	FCz, Cz, CPz	FC4, FC6, C4, C6, CP4, CP6
Posterior	P3, P5, P7, PO3, PO5, PO7	Pz, POz, Oz	P4, P6, P8, PO4, PO6, PO8

In the analysis of the general linear model, data were adjusted by the Bonferroni correction. All *p*-values reported below were adjusted with the Greenhouse–Geisser correction when the degree of freedom in the numerator was larger than 1. The reported eta squared (η^2^) was used to measure the effect size for ANOVAs (Olejnik and Algina, 2003).

### Results

#### Behavioral Results

Behavioral results were analyzed in R studio 3.5.1 software (R Development Core Team, 2012). Given the poor performance of participants would influence the results, the data were deleted for the trials in which the RTs were shorter than 500 ms or longer than 3SD above the overall average. As a result, 9.2 and 7.68% of the collected data were discarded in Tasks 1 and 2, respectively. The accuracy (ACC) and reaction time (RT) were recorded by computer automatically. In Tasks 1 and 2, the participants responded with a mean accuracy of 91.55% (*SD* = 0.258) and 92.45% (*SD* = 0.25), respectively, indicating that they followed the instructions and attended to the stimuli carefully. The mean reaction time in Tasks 1 and 2 was 1,136.75 ms (*SD* = 339.5) and 1,137.75 ms (*SD* = 36), respectively. A main effect of verb type was found in both tasks (Task 1: β = 0,112, *SE* = 0.041, *df* = 70.275, *t* = 2.73, *p* < 0.01; Task 2: β = 0.155, *SE* = 0.036, *df* = 39.450, *t* = 4.361, *p* < 0.01), i.e., the participants responded faster in action verbs condition than in state verbs condition.

#### Electrophysiological Results

**Figure 3:**
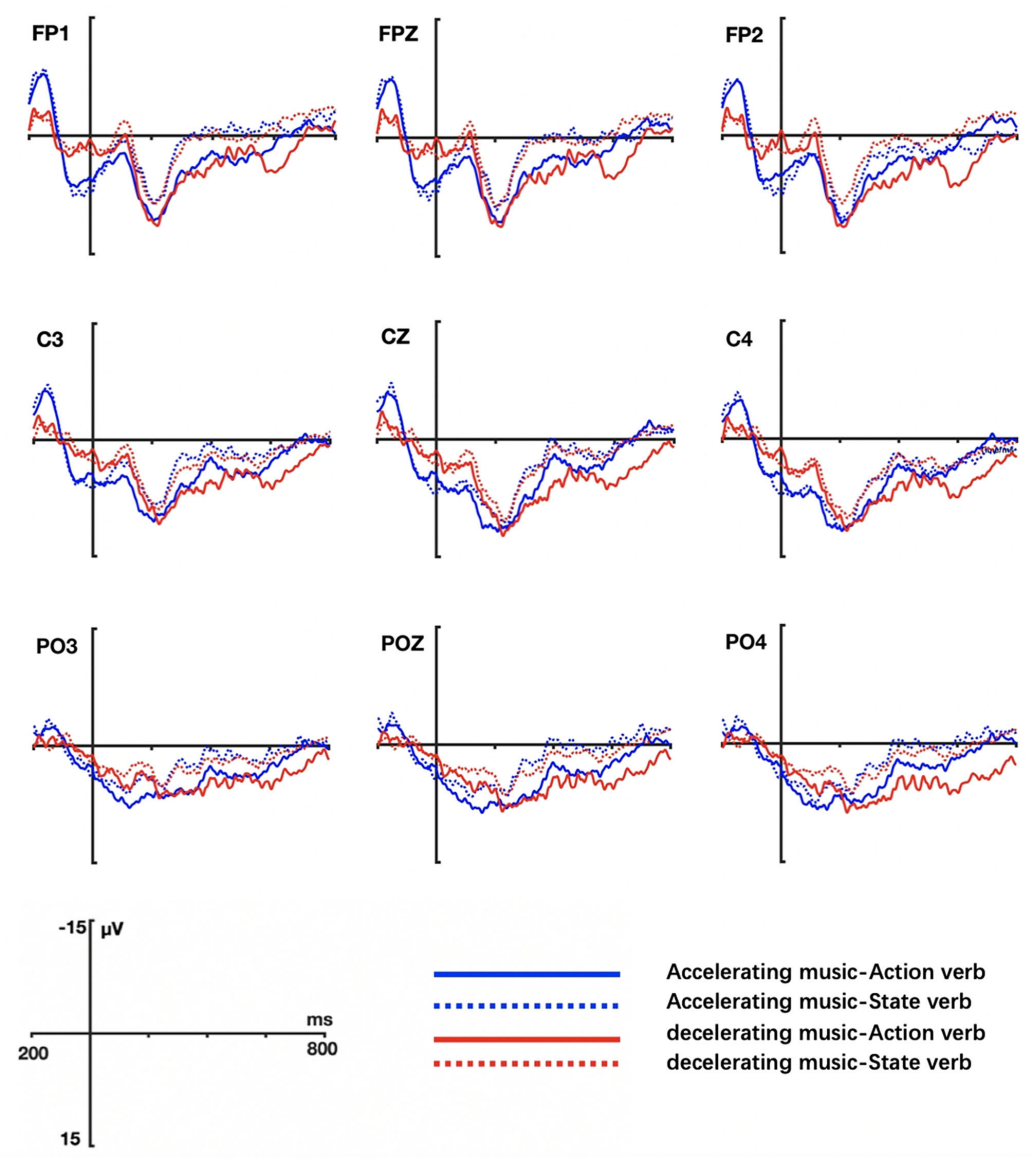
Grand ERP averages elicited by action and state verbs in tempo-with-change music priming conditions (Task 1).

**Figure 4:**
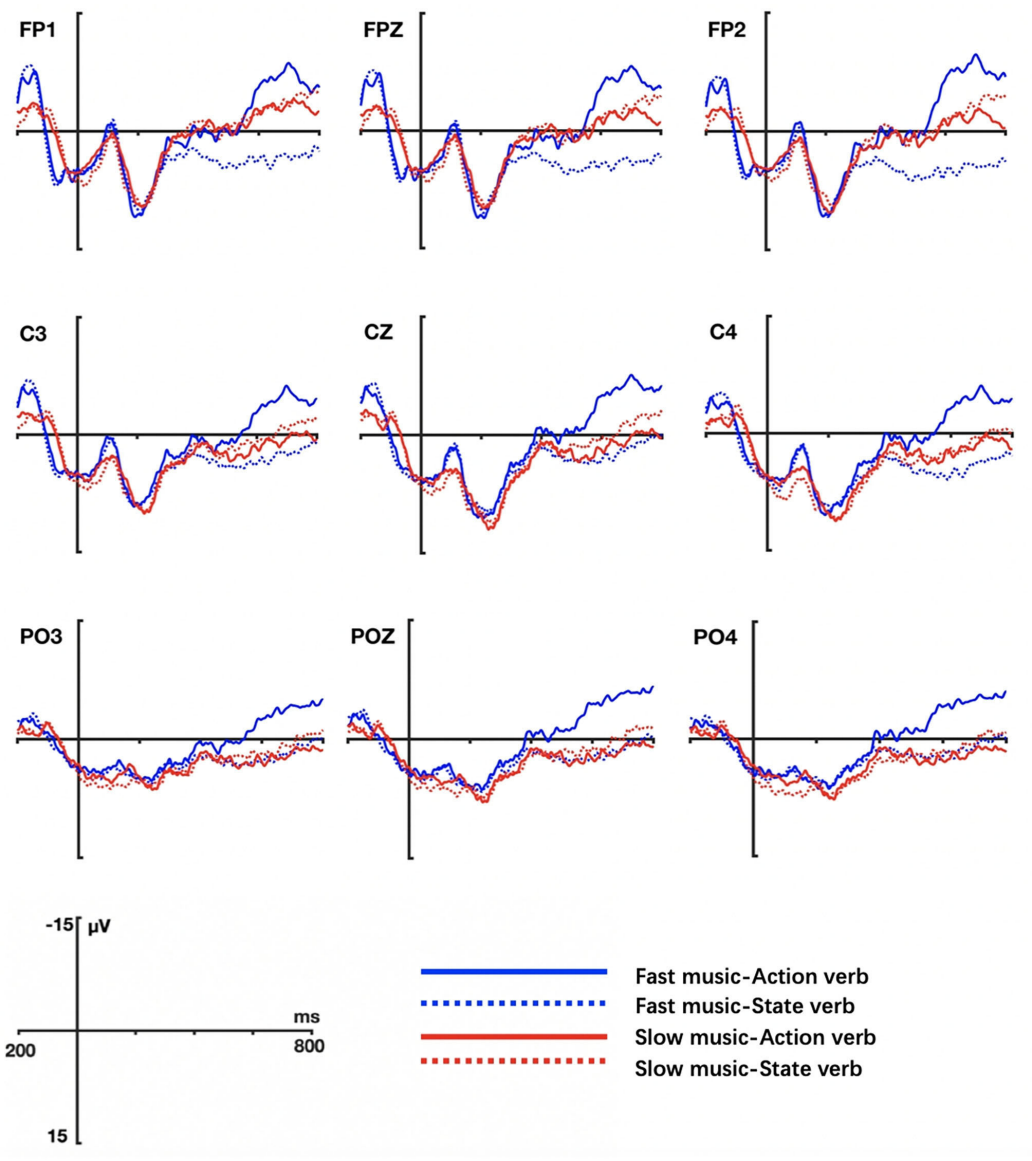
Grand ERP averages elicited by action and state verbs in tempo-without-change music priming conditions (Task 2).

The grand averaged ERP waveform elicited by actions verbs and state verbs in different music conditions are shown in Figure 3 (Task 1) and Figure 4 (Task 2) respectively.

**Figure 5:**
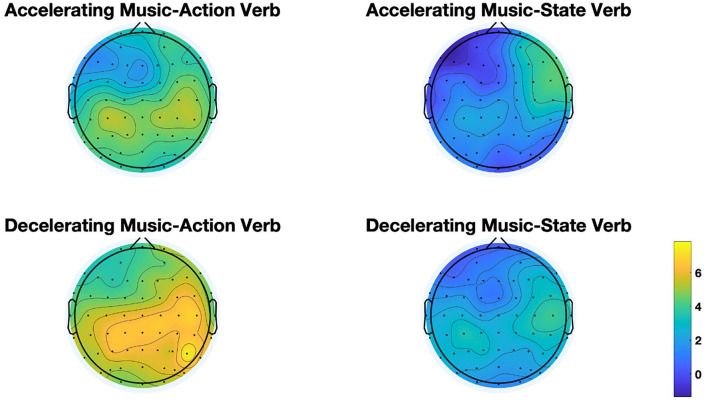
Scalp topographic maps of N400 across four conditions in Task 1.

In Task 1, the ERP waves to action verbs and state verbs diverged at ~250 ms after the target verb onset and reached maximality at 400 ms and afterward lasted one more 100 ms (at 500 ms) (as shown in Figure 3). Statistical analysis showed that there was a significant main effect of verb (lateral electrodes: *F* = 7.557, *p* = 0.009, η^2^ = 0.17) in that state verbs elicited larger N400 amplitudes than action verbs independent of music condition. In addition, there occurred a significant interaction between music and verb type (middle electrodes: *F* = 10.593, *p* = 0.002, η^2^ = 0.223). Further simple effect test on verbs showed that state verbs compared to action verbs evoked enhanced N400 amplitudes in both accelerating music (lateral electrodes: *F* = 9.309, *p* = 0.004, η^2^ = 0.201) and decelerating music conditions (middle electrodes: *F* = 10.591, *p* = 0.002, η^2^ = 0.223). Another simple effect test on music revealed that action verbs induced larger amplitudes in accelerating music condition than in decelerating music condition around the anterior region (middle electrodes: *F* = 5.108, *p* = 0.03, η^2^ = 0.121) (as exhibited in Figure 5). Significant interactions were observed between verb and hemisphere (*F* = 4.132, *p* = 0.049, η^2^ = 0.100) but no significant interactions were found between music and hemisphere (*F* = 0.16, *p* = 0.692, η^2^ = 0.004), music and region (lateral: *F* = 2.495, *p* = 0.119, η^2^ = 0.063; middle: *F* = 1.347, *p* = 0.252, η^2^ = 0.036), and between verb and region factor (lateral: *F* = 2.178, *p* = 0.142, η^2^ = 0.056; middle: *F* = 2.349, *p* = 0.132, η^2^ = 0.060). Moreover, there were not three-way interactions among music, verb, hemisphere (*F* = 2.455, *p* = 0.126, η^2^ = 0.062) and music, verb, region factors (lateral: *F* = 2.518, *p* = 0.112, η^2^ = 0.064; middle: *F* = 2.506, *p* = 0.117, η^2^ = 0.063).

To sum up, electrophysiological results of task 1 showed that state verbs elicited a larger N400 than action verbs, independent of music type over the anterior regions at the array of analyzed electrodes. Moreover, action verbs relative to state verbs were more sensitive to music type, i.e., action verbs elicited a larger amplitude in accelerating music conditions than in decelerating music conditions during processing action verbs.

In Task 2, the results were quite different. Statistical analysis showed that the main effect of verb type was not significant at both lateral electrodes (*F* = 0.018, *p* = 0.893, η^2^ = 0.000) and middle electrodes (*F* = 0.045, *p* = 0.833, η^2^ = 0.001), indicating that action verbs and state verbs were not so differently processed when primed by the music without tempo changes (fast or slow music). However, there was a significant interaction between music type and verb in the midline (*F* = 7.985, *p* = 0.008, η^2^ = 0.177) and lateral electrodes regions (*F* = 9.22, *p* = 0.004, η^2^ = 0.199). The simple effect test on verb indicated that action verbs elicited larger N400 amplitudes than state verbs in both fast music condition (lateral electrodes: *F* = 6.422, *p* = 0.016, η^2^ = 0.148) and slow music condition (lateral electrodes: *F* = 5.887, *p* = 0.02, η^2^ = 0.137; middle electrodes: *F* = 5.682, *p* = 0.022, η^2^ = 0.133). There were no significant interactions between music and electrode position factors and between verb and electrode position factors, both for lateral (music × hemisphere: *F* = 0.101, *p* = 0.752, η^2^ = 0.003; verb × hemisphere: *F* = 1.650, *p* = 0.687, η^2^ = 0.004; music × region: *F* = 0.237, *p* = 0.643, η^2^ = 0.006; verb × region: *F* = 0.749, *p* = 0.418, η^2^ = 0.02) and middle electrodes (music × region: *F* = 0.749, *p* = 0.418, η^2^ = 0.02; verb × region: *F* = 0.072, *p* = 0.889, η^2^ = 0.002). No three-way interactions among music, verb, hemisphere (lateral: *F* = 3.712, *p* = 0.062, η^2^ = 0.091) and music, verb, region factors (lateral: *F* = 0.323, *p* = 0.594, η^2^ = 0.009; middle: *F* = 0.415, *p* = 0.662, η^2^ = 0.011) were found.

In brief, the music without tempo changes affected the processing of verbs. Contrary to the situation in Task 1, action verbs induced a larger N400 than state verbs in both music conditions.

### Discussion

Study 1 intended to investigate how Chinese verbs were processed in different music priming conditions provided that verbs and music take on the shared motion concepts. The ERP data revealed that the effects of different music tempo types were varied. The N400 was elicited by state verbs in Task 1 but by action verbs in Task 2, implying that music with tempo changes facilitated the processing of action verbs but music without tempo changes inhibited the processing of action verbs. That is, change-in-tempo music was more related to the motion concept and triggered the cognition of action verbs as a result. This result partly justifies the first prediction that music with tempo changes can facilitate the processing of verbs.

Unexpectedly, the motion congruency effect by N400 was not found in the two tasks. In different music conditions, action verbs in music with strong motion information conditions (accelerating music in Task 1 and fast music in Task 2) induced larger ERP amplitudes, contrary to our second prediction that processing congruent pairs (accelerating music-action verbs; fast music-action verbs) should be easier. This result was somewhat comparable to the word-repetition situation in which the resulting ERP wave of a word becomes stronger when it is primed on its own than by a different but relevant word (Michael, 1985). Here, we may assume that it is the repetition of motion concepts shared by music and action verb that leads to the increased ERP amplitudes relating to the processing of action verbs. Another related possibility is that establishing the congruency between music and verbs is similar to the music-picture pairs manipulations in Hedger et al. (2013) but more difficult due to the inherent greater complexity in words than in pictures, for the motion concept is encoded directly by pictures but indirectly by words.

The results also demonstrate that the processing patterns between action verbs and state verbs vary with music types. The attenuated N400 amplitudes of action verbs suggest that the processing of action verbs is easier and compatible with the third prediction. In addition, the significant difference between music conditions observed only in action verbs suggests that the sub-types of music with tempo changes only affect action verbs but not state verbs. That is, action verbs seem more sensitive to music conditions. In our study, state verbs elicited enhanced N400 amplitudes, confirming Muraki et al.'s (2020) finding that non-bodily state verbs elicited larger N400 amplitudes than concrete verbs in a syntactic classification task. The motion information in action verbs appears to be simulated more easily than in state verbs due to the more motion information they share.

In brief, the different effects of music tempo types on different verbs verify the idea that people tend to analogically understand the motion concept in music and language by virtue of the acoustic features and linguistic meaning. In this process, bodily motion experience is supposed to be activated as soon as the music and the verbs with high motion information are presented. Yet this experience does not bring about the absolutely similar activation in music and words because they are two different categories and hence distinctly encoded with regard to motion concept.

## Study 2

Verbs and nouns are two important word classes in Chinese. As revealed in Study 1, verbs have motion attributes, but in folk cognition, verbs are assumed to contain more dynamic information while the majority of nouns contain more static information. Neurophysiological studies show that verbs and nouns are differently processed (e.g., Pulvermüller et al., 1999). In addition, nouns (just like verbs) can be subdivided into many subcategories, among which is the dichotomy of animate nouns and inanimate nouns in terms of the trait (±ANIMACY). In linguistics, it is generally agreed that animate nouns are more associated with action than inanimate nouns, for the former is used to signify objects in motion whereas the latter signals entities at rest in general (Weckerly and Kutas, 1999). On this account, Study 2 was conducted to compare how to animate nouns and inanimate nouns were processed in different priming-music conditions, and the music pieces were just the same sets as adopted in Study 1.

### Methods

Just like Study 1, Study 2 adopted the cross-modality concept priming paradigm by using music to prime nouns. The experiment was comprised of two tasks, i.e., Task 3 testing nouns primed by music with tempo changes (accelerating music; decelerating music) and Task 4 testing the nouns primed by music without tempo changes (fast music; slow music). All the targets were animate and inanimate nouns, with half for each. The priming stimuli and target stimuli constituted four congruency conditions, with two congruent pairs (accelerating music- animate nouns, decelerating music-inanimate nouns) and two incongruent pairs (accelerating music-inanimate nouns, decelerating music-inanimate nouns). The whole experiment lasted around 14 min.

#### Participants

The participants were those who had participated in Study 1. After the experiment, the data of one participant were discarded due to the EEG equipment malfunctioning. Another participant's data were excluded due to her failure to follow the experiment instructions. As a result, there thirty-eight participants' data were kept for analysis. All the participants signed the consent form prior to the experiment and were paid for their participation at the terminal of the experiment.

#### Stimuli Construction

The priming stimuli of Study 2 were identical to those adopted in Study 1 and the target stimuli were replaced by nouns (from verbs). Priming stimuli in Tasks 3 (accelerating music and decelerating music) and 4 (fast music and slow music) repeated the music motifs in Tasks 1 and 2, respectively, including ten music motifs in each condition. Forty animate nouns and forty inanimate nouns selected from the CCL corpus were used as target stimuli in the two tasks. Each task involved 20 animate nouns and 20 inanimate nouns. No nouns were repeated in the two tasks. All the nouns were concrete and with high frequency, excluding emotional words. The animate nouns were featured by agentivity and high probability in motion or action. For example, the noun 豹子 “*baozi*/ leopard” has the ability to perform an action (e.g., running) and the abstract motion concept is encoded in this concrete entity. By contrast, inanimate nouns were characterized by null agentivity and high staticity, such as 沙发 “*shafa/*sofa” and 雕像 “*diaoxiang*/statue”, which often serve as the undergoer to receive action(s).

#### Stimuli Pre-tests

As in Study 1, in order to manipulate the motion concept of nouns as well as their relatedness with music tempos, we made the pre-tests on the basis of seven-point scales. The steps were identical to those in Study 1.

In the motion attribute test, the mean score of the animate nouns was 1.952 (*SD* = 0.248) and the inanimate nouns −2.114 (*SD* = 0.245), suggesting that the participants could clearly distinguish animate nouns with motion concept from inanimate nouns without motion concept. An independent sample *t*-test showed that the two classes of nouns were significantly different from each other with regard to motion information [*t*_(78)_ = 73.734, *p* < 0.01].

In the imaginability test, the mean scores of the animate nouns and inanimate nouns were 2.252 (*SD* = 0.241) and 2.197 (*SD* = 0.198), respectively. The independent *t*-test showed that there was no significant difference in their imaginability [*t*_(78)_ = 1.114, *p* = 0.269].

In the relatedness test, four conditions (pairs) and two relatedness relations (congruent or incongruent) were formed in Study 2, comprising Tasks 3 and 4. Congruent pairs were those with congruent motion information: accelerating (or fast) music—animate noun pairs; decelerating (or slow) music—inanimate noun pairs. On the opposite, incongruent pairs were those with incongruent motion information: accelerating (or fast) music—inanimate noun pairs; decelerating (or slow) music—animate noun pairs. Results showed that the mean scores of the congruent pairs in Tasks 3 and 4 were 1.967 (*SD* = 0.226) and 2.010 (*SD* = 0.248) respectively whereas the mean score of the incongruent pairs in Tasks 3 and 4 are −1.880 (*SD* = 0.201) and −1.708 (*SD* = 0.191), respectively, suggesting that most adults can perceptively (i.e., *via* auditory and visual embodiment) establish the relations between music and nouns. Further comparison showed that the related pairs were strikingly different from the unrelated pairs in both Tasks 3 [*t*_(38)_ = 56.939, *p* < 0.01] and 4 [*t*_(38)_ = 53.101, *p* < 0.01]. The two classes of nouns' stroke number were manipulated (animate noun: *M* =17.700, *SD* = 4.767; inanimate noun: *M* = 16.875, *SD* = 4.598), with the independent *t*-test showing that they were not significantly different [*t*_(78)_ = 0.788, *p* = 0.433].

According to the rating results, 40 pairs in each task (with 10 pairs in each condition) were chosen as the formal stimuli for the experiment afterward. Items and conditions were counterbalanced in each task, controlled *via* E-prime 3.

#### Procedures

The procedure was identical to Study 1.

#### EEG Recordings and Preprocessing

The recording methodologies and the data preprocessing were identical to those in Study 1.

#### ERP Data Analysis

The data analysis of Study 2 followed the same procedure as in Study 1.

### Results

#### Behavioral Results

To follow the general norm for experimental studies, we deleted the data with RTs shorter than 500 ms or longer than 3 SD above the overall average. As a result, 3.82 and 4% of the collected data were discarded in Tasks 3 and 4, respectively. Behavioral results showed that the participants responded with a mean accuracy of 94.44% (*SD* = 0.211) and 93.8% (*SD* = 0.181) for the two tasks, respectively, indicating that they followed the instructions and attended to the stimuli carefully. The mean reaction time of Tasks 3 and 4 was 1,019.75 ms (*SD* = 344.25) and 1,051.25 ms (*SD* = 322.25), respectively. A significant main effect of noun were observed in the two tasks, in which participants had a faster response to animate nouns than to inanimate nouns (Task 3: β = 0.101, *SE* = 0.027, *df* = 28.043, *t* = 3.726, *p* = 0.001; Task 4: β = 0.119, *SE* = 0.023, *df* = 31.83, *t* = 5.108, *p* < 0.01), suggesting animate nouns seem to be easier to process than inanimate nouns.

#### Electrophysiological Results

**Figure 6:**
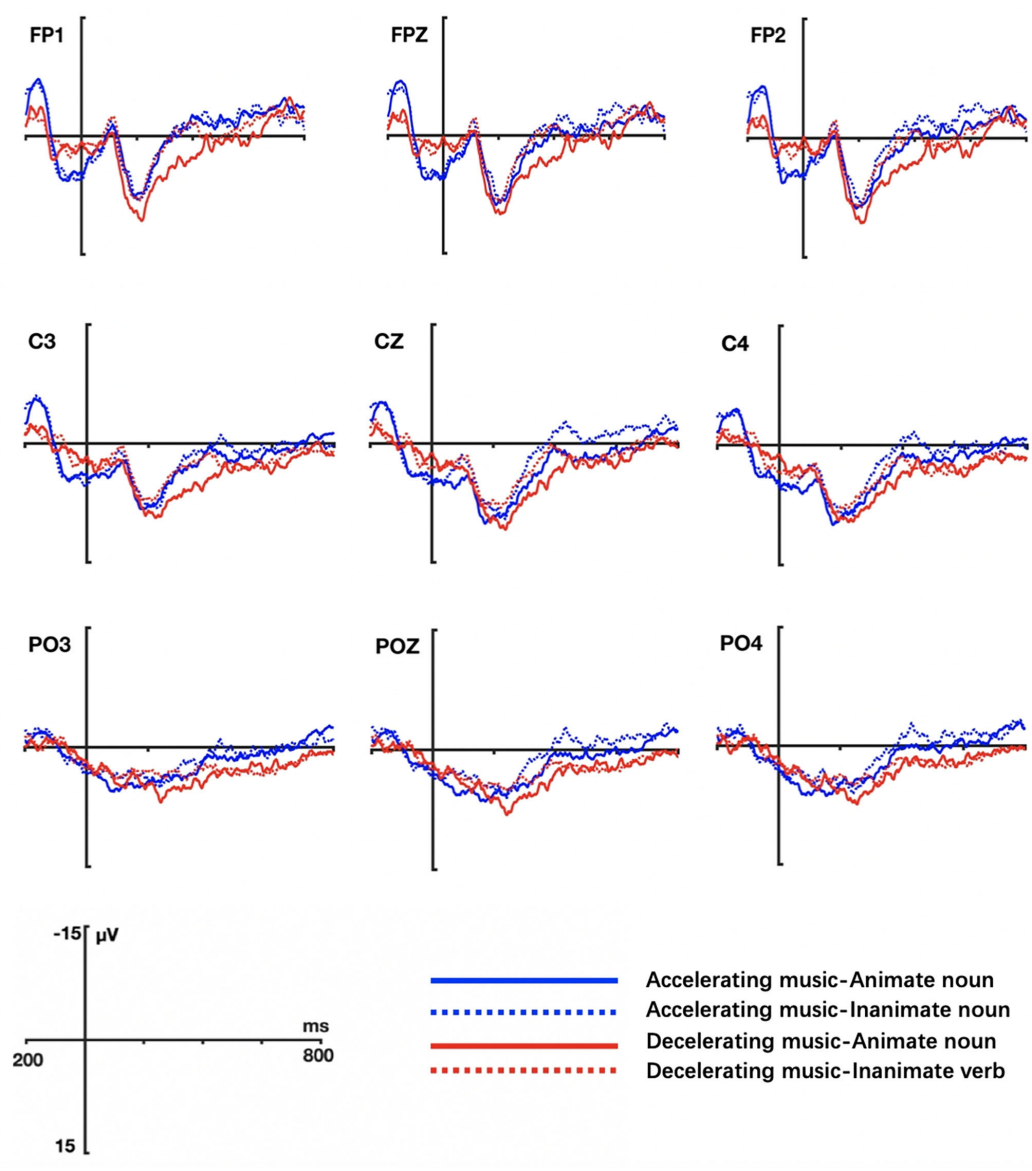
Grand ERP averages elicited by animate and inanimate nouns in tempo-with-change music priming conditions (Task 3).

**Figure 7:**
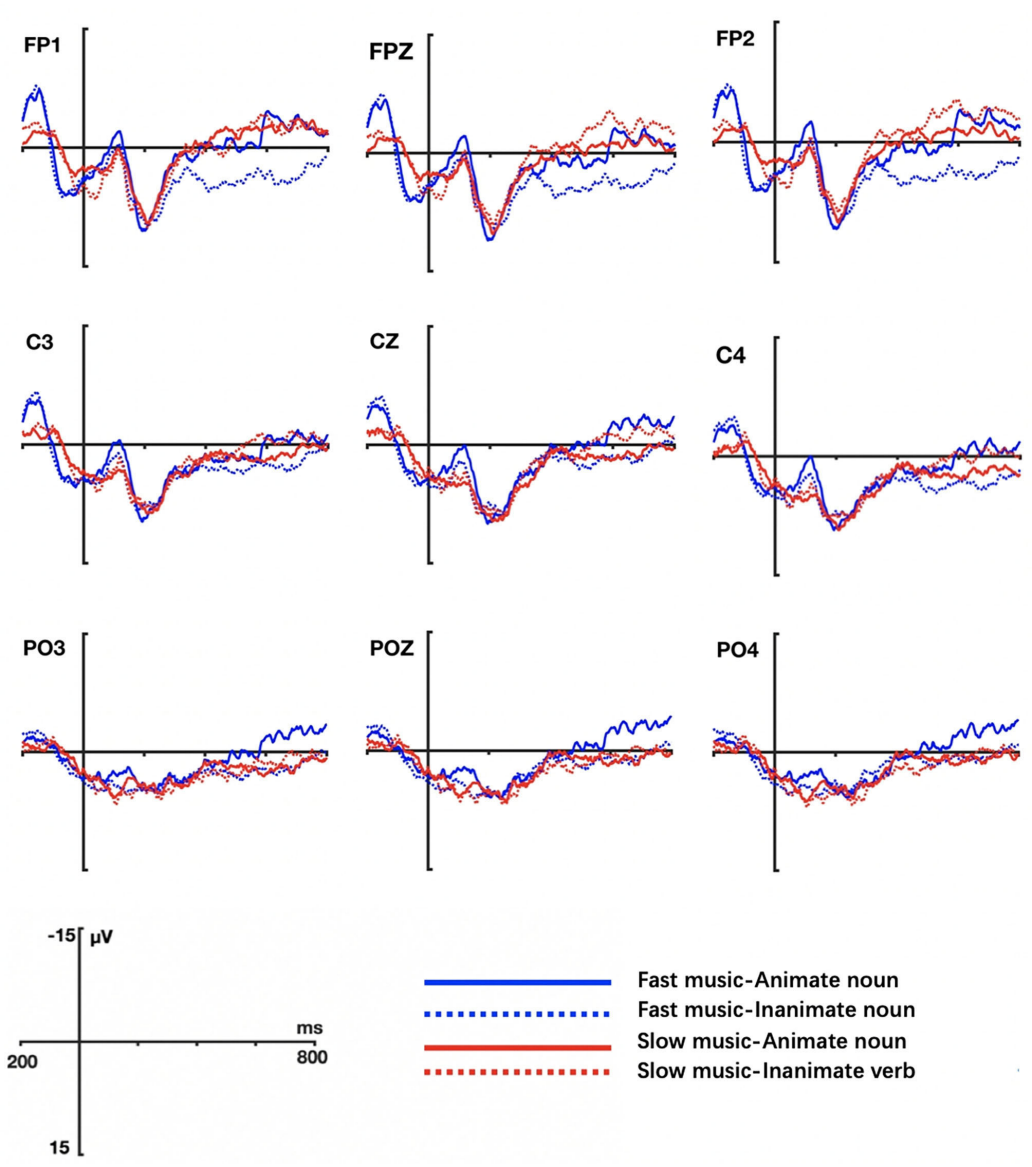
Grand ERP averages elicited by animate and inanimate nouns in tempo-without-change music priming conditions (Task 4).

The grand averaged ERP waveform elicited by animate nouns and inanimate nouns in different music conditions were shown in Figure 6 (Task 3) and Figure 7 (Task 4).

The results of Task 3 were comparable to those of Task 1 in Study 1. Visual inspection showed that larger negativity amplitudes were induced by inanimate nouns, which started from 250 ms and peaked at 400 ms, and lasted to 500 ms (as illustrated in Figure 6). ANOVA was utilized to test the ERPs of a 2 music (Task 1: accelerating music, decelerating music; Task 2: fast music, slow music) × 2 noun (animate noun, inanimate noun) × 2 hemispheres (left hemisphere, right hemisphere) × 3 regions (anterior, central, posterior) in this experiment.

**Figure 8:**
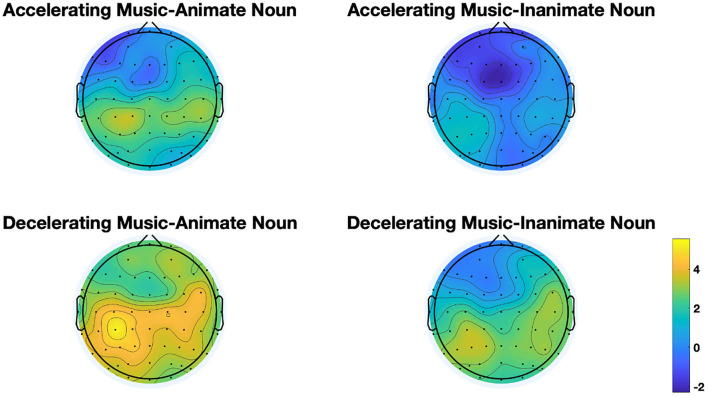
Scalp topographic maps of N400 across the four conditions in Task 3.

In the noun conditions, the analysis showed an observable effect of noun only at lateral electrodes (*F* = 17.413, *p* = 0.000, η^2^ = 0.32). To be precise, compared with animate nouns, inanimate nouns incurred increased N400 amplitudes over the anterior and central regions (as exhibited in Figure 8), similar to Zhou et al.'s study (2015), in which the target stimuli were non-verbal pictures. Besides, we observed a main effect of music in middle electrodes regions (*F* = 16.843, *p* = 0.000, η^2^ = 0.313). No interactive effects were found between music and nouns (lateral: *F* = 0.934, *p* = 0.340, η^2^ = 0.025; middle: *F* = 1.97, *p* = 0.659, η^2^ = 0.005). To further explore the influence of different music conditions on animate and inanimate nouns, we conducted a simple-effect test, with results showing that inanimate nouns induced greater N400 than the animate nouns in accelerating music conditions (lateral: *F* = 13.011, *p* = 0.001, η^2^ = 0.26) and decelerating music conditions (middle: *F* = 5.024, *p* = 0.031, η^2^ = 0.12). A simple test on the effect of music on nouns further revealed that only action verbs triggered an intensifying N400 in accelerating music than in decelerating music (lateral: *F* = 5.574, *p* = 0.024, η^2^ = 0.131), but inanimate nouns did not show conspicuous processing difference in the two priming-music conditions. In addition, there were significant interactions between noun and region factor (*F* = 9.931, *p* = 0.002, η^2^ = 0.212) and between noun and hemisphere factor (*F* = 7.572, *p* = 0.009, η^2^ = 0.17) in lateral electrodes. No marked three interaction among music, noun, hemisphere (lateral: *F* = 2.656, *p* = 0.112, η^2^ = 0.067) and music, noun, region (lateral: *F* = 0.138, *p* = 0.738, η^2^ = 0.004; middle: *F* = 0.634, *p* = 0.451, η^2^ = 0.017) were triggered.

In Task 4, there was no main effects in noun type and music type both in lateral (Noun: *F* = 0.002, *p* = 0.966, η^2^ = 0.000; Music: *F* = 0.032, *p* = 0.858, η^2^ = 0.001) and middle electrodes (Noun: *F* = 0.054, *p* = 0.818, η^2^ = 0.001; Music: *F* = 0.026, *p* = 0.874, η^2^ = 0.001). No significant interactions were found between music without tempo changes and nouns (lateral: *F* = 0.213, *p* = 0.647, η^2^ = 0.006; middle: *F* = 0.725, *p* = 0.400, η^2^ = 0.019). These results suggest that the N400 amplitudes induced by the two types of nouns did not differ significantly, nor did those induced by the two types of music. There were no significant interactions between noun and electrode regions factors (lateral: noun × region: *F* = 0.158, *p* = 0.763, η^2^ = 0.004; noun × hemisphere: *F* = 0.09, *p* = 0.926, η^2^ = 0.000; middle: noun × region: *F* = 0.839, *p* = 0.395, η^2^ = 0.022) and music and electrode regions factors (lateral: music × region: *F* = 2.608, *p* = 0.112, η^2^ = 0.066; music × hemisphere: *F* = 0.892, *p* = 0.351, η^2^ = 0.024; middle: music × region: *F* = 0.713, *p* = 0.426, η^2^ = 0.018). However, there occurred significant interactions among music, noun, hemisphere (lateral: *F* = 10.663, *p* = 0.002, η^2^ = 0.224) and among music, noun, region (middle: *F* = 0.479, *p* = 0.031, η^2^ = 0.112).

### Discussion

This study investigated the processing of nouns primed by different music tempo types. As expected, the results of Tasks 3 and 4 displayed that music with tempo changes influenced the processing of nouns while music without tempo changes did not, which is compatible with Hedger et al.'s (2013) study. This result demonstrated that tempo changes in music were more related to motion concept and triggered the cognition of nouns as a result. Therefore, participants could metaphorically establish the relations of the motion concept between this type of music and nouns on the basis of embodiment. However, this result was not observed in Task 4, suggesting that music without tempo changes can not differentiate between animate nouns and inanimate nouns, as the N400 amplitudes induced by the two types of nouns were not strikingly different. This finding appeared to justify an important recognition that sensorimotor experience was activated in music with tempo changes and nouns in some way, indexed by N400, consistent with our first prediction. Furthermore, this result extends the finding of Task 1, i.e., music with tempo changes influences not only action verbs but also animate nouns. Consequently, we infer that the participants can hardly map the relations between music without tempo changes and nouns (or verbs), as indicated by the absence of different N400 amplitudes and that motion information may not be an integral component of this music type in question, at least off stage in the processing of nouns.

Roughly like the case in Study 1, the processing advantages of congruent pairs failed. To be specific, the increased N400 amplitudes elicited by animate nouns was larger in accelerating music condition than in decelerating music condition, inconsistent with our second prediction. So to speak, the similar effect of music with tempo changes occurred on action verbs in Task 1 and on animate nouns in Task 3. In terms of the word-repetition effect (Michael, 1985) mentioned in Study 1, we assumed that it was the re-occurrence of motion information that led to the N400 amplitudes of animate nouns, for the information was shared by accelerating music and animate nouns.

The electrophysiological results showed that the greater N400 was induced by inanimate nouns (in Task 3), indicating the greater difficulty in processing inanimate nouns vs. animate nouns. Furthermore, in Task 3, the results that music with tempo changes could affect only animate nouns but inanimate nouns, implying that the motion concept correlation between the music with tempo changes and animate nouns is easier to establish. As a result, the sensorimotor experience can be harder to activate during the processing of inanimate nouns, or inanimate nouns are not conceptually related to the motion concept at all, favoring the third prediction.

Above all, Study 2 shows that the embodied experience can be activated in music with tempo changes and animate nouns, indexed by N400. The processing of animate nouns is easier to process than inanimate nouns due to the more motion information they have, favoring our prediction three. There is no motion congruency effect of N400 in music and language, disfavoring the second prediction.

## General Discussion

The two ERP studies aim to investigate how verbs and nouns are processed under two music priming conditions by means of a cross-modality priming paradigm (music listening followed by words' visual reading). Results show that music types influence different classes of words uniquely, action verbs and animate nouns contrast with state verbs and inanimate nouns in processing patterns, and the motion concept as a high-level meaning can be well-established between music tempos and words on the embodied metaphor basis, indexed by N400. The processing of words varies with music tempos, which is not determined by the congruency between music and words but influenced by the properties of music tempos and words.

### Tempo-Change Music Affecting Action Verbs and Animates Nouns

The experimental results show that music with tempo changes facilitates the processing of action verbs and animate nouns as revealed by the attenuated ERP amplitudes of the words, consistent with our first prediction. Yet as the priming stimuli are transformed into music without tempo changes, verbs show just the opposite pattern while nouns are not differently affected. The different N400 patterns between the two tasks (Task 1 vs. 2; Task 3 vs. 4) are supposed to result from the attributes of music tempos, suggesting that motion concept is encoded to different degrees in both music and words, i.e., more motion information in music with tempo changes and action verbs and animate nouns while less motion information in music without tempo changes and state verbs and inanimate nouns. According to Hedger et al. (2013), music tempos are closely related to the different properties of speed representations. For accelerating and decelerating music, the number of notes becomes either more or less dense within a single music excerpt, wherein individuals can perceive and expect its speed changes. That is to say, music with tempo changes has strong energy (Eitan and Granot, 2006), hence embracing strong-motion motivation. In statically fast and slow music motif conditions, however, tempos within a music motif have no temporal changes. Here, “fast” and “slow” can be labeled only by comparing one music excerpt with the other. Consequently, compared with statically fast and slow music, the properties of accelerating and decelerating music give people certain clues to identify dynamic changes and anticipate motion changes.

In parallel to the music with tempo changes, action verbs and animate nouns are encoded with more motion information than state verbs and inanimate nouns, giving rise to the reduced N400 effect in words as a result. By contrast, the music without tempo changes is associated with the state rather than motion in concept, and its lack of motion fails to trigger the motion concept in words, resulting in no ERP amplitudes in verbs and nouns as well as their subcategories. That may also explain why people cannot well-establish the relations between music without tempo changes and words (verbs and nouns), to be elaborated below.

### Motion Concept-Based Congruency Between Music and Words

In the experiments, participants' responses were not influenced by compatibility between music with or without tempo changes and words (verbs and nouns). The unexpected results that congruent pairs elicited enhanced N400 in Tasks 1, 2, and 3 but both congruent and incongruent pairs induced no amplitude difference in Task 4 to suggest that accelerating music and fast music inhibit the processing of action verbs and animate nouns, seemingly contradictory to Zhou et al.'s (2015) study that incongruency between music and pictures enhanced larger N400. The following makes an attempt to provide several possible explanations for the issue.

The first is due to the greater difficulty of the motion concept in verbs and nouns than in non-verbal stimuli (e.g., pictures). In Hedger et al.'s (2013) study, accelerating music and decelerating music better-primed pictures in motion and at rest, respectively. Compared with pictures, words seem more difficult to have the motion concept extracted, for the decoding of the motion concept in words is involved a more complex process. In general, visual pictures involve a visual perception system at a low level (Kamio and Toichi, 2000; Ostarek and Hüettig, 2017), whereas language processing is an advanced activity of human cognition. According to the spreading activation modal (Collins and Quillian, 1970) that each word related to an entry in the mental lexicon may be activated as one hears a word. We assume that decoding the motion concept of words, one of the lexical aspects of the mental lexicon, may be influenced by other “competitors” like literal meaning and figurative meaning. Compared with language, an image depicted in the scene is more salient and thus restricts other alternatives and “competitors” that may be activated in the mental lexicon (Altmann and Kamide, 1999) during language comprehension. By contrast, the motion concept in the language is not directly encoded and becomes slowly extracted or decoded during linguistic comprehension. As a result, establishing the congruency seems more difficult between music and words than between music and pictures.

The second explanation is related to the word repetition effect. It is discovered that repeated words display more negative-going ERP amplitudes than non-repeated but related words (Michael, 1985). In our experiments, both words (action verbs and animate nouns) and music (accelerating music and fast music) have high motion energy and attributes. Probably, it is the repeated motion information by action verbs and animate nouns that evoked the larger N400. If it is so, the repetition effect is not confined to concrete words with the same form but can be extended to the abstract notion like the motion concept implied in words.

The third reason may be due to the relatively small number of materials adopted in our experiments. Although our studies adopted the same number of musical and word materials as Hedger et al. (2013), the trials are actually less than those in many purely linguistic ERP experiments, which may partly explain the discrepancy between linguistic priming paradigm (within the same domain) and music-linguistic priming paradigm (across domains).

### Processing Patterns Differentiating Between Word Types

The two studies flag the different processing patterns of the four types of words (action verbs, state verbs, animate nouns, and inanimate nouns). As tempo-change music serves as the prime, action verbs and animate nouns show easier processing patterns than state verbs and inanimate nouns, respectively, both in accelerating music and decelerating music condition, adding evidence to the view that motion concept is metaphorically established across categories *via* our bodily experience. From the words' encoding perspective, action verbs and animate nouns are associated with more motion information while state verbs and inanimate nouns are often deemed as words with low motion (Muraki et al., 2020). State verbs and inanimate nouns induce larger N400 amplitudes when primed by music with tempo changes but do not interact with the music sub-types, suggesting that they convey less motion information than action verbs and animate nouns. In light of embodiment theories, semantic processing is wholly reliant on sensorimotor experience (Lakoff and Johnson, 1999, p. 151; Glenberg and Kaschak, 2002), our bodily experience non-technically. Likewise, the comprehension of the motion concept in words also cannot be separated from embodied experience. According to Grossman et al. (2002), there is some degree of sensorimotor representation during motion-verb processing while the sensorimotor systems are not involved in the abstract verb representation. Therefore, compared with state verbs, action verbs are more prominent in motion information, which may yield the easier processing of action verbs relative to state verbs in our studies. That is, verbs with more motion information (e.g., action verbs) can be better understood with the help of our previous bodily experience. The processing difficulty of inanimate nouns in our motion-related task extends the previous studies that inanimate nouns in subject position induced larger N400 amplitudes as they are not the ideal actor to perform action relative to animate nouns (Dahl, 2008; Philipp et al., 2008). This finding is consistent with Philipp et al.'s (2008) claim that animate nouns as the typical agent have the ability to perform action whereas inanimate nouns often serve as the undergoer and with Muraki et al.'s (2020) finding that abstract non-bodily state verbs elicited greater N400 amplitudes (at frontocentral electrodes) relative to abstract mental state and concrete verbs in a syntactic classification task.

In no-tempo-change music conditions, only verbs show their differences in their subcategories while the processing of nouns does not vary with fast and slow music conditions, suggesting verbs and nouns are different distinguished by this kind of music. Cognitively, verbs and nouns represent dynamic and static characteristics in image schema, respectively (Li, 2007), taking on different neural basis. From the perspective of processing, verbs are related to the motor cortex and the frontal lobe (near the motor cortex), and nouns are associated with the visual cortex and temporal occipital area (near the visual center) (Pulvermüller et al., 1999). That is to say, verbs involve more motion information than nouns in processing. In our studies, verbs are more sensitive to music without tempo changes than nouns probably owing to the sensorimotor experience that has been activated. A similar difference also finds expressions in the loci involved in processing the two classes of words, i.e., nouns are mainly distributed in the anterior and central regions whereas verbs only activate the anterior regions, indicating verbs are more frontally-distributed in processing. In their sub-categories, action verbs and animate nouns induced larger amplitudes both in fast and slow music conditions suggesting that music without tempo changes inhibited these kinds of verbs. Besides, the N400 amplitudes of animate nouns and inanimate nouns are not affected by music without tempo changes. These reversed results demonstrate that music with tempo changes fail to facilitate the processing of verbs and nouns, probably owing to the different role of music tempo types, as illustrated in section Motion Concept-Based Congruency Between Music and Words.

### N400 Indexing Motion Concept by Music and Words

In general, N400 is an electrophysiological index responsible for semantic violation in both languages (e.g., Kutas and Hillyard, 1980; Kutas and Federmeier, 2011) and music (e.g., Koelsch et al., 2004). Moreover, the N400 effects basically keep constant in language conditions (in which target words are preceded by a sequence of words or sentences) and in music conditions (in which target words are preceded by musical excerpts) (Koelsch, 2011). Music is different from words in meaning representation, but they share the same conceptual meaning—the motion concept, which is reliant on sensorimotor experience. In the present study, the motion concept as the extra-musical meaning in music was perceived auditorily but the metaphorical meaning in words was understood visually. The result that action verbs and animate nouns can well-represent the motion concept under tempo-change music conditions demonstrates that the motion concept is semantic in nature, and the resulting N400 is believed to be the electrophysiological indicator of the shared motion by music and words. Our studies are supportive of the hypothesis that music and language share the neural mechanism (N400) of meaning processing.

According to the perceptual symbol's hypothesis (Barsalou, 1999), the storage of perceptual symbols in memory based on perception is extracted from experience (e.g., basic perception like color, emotion, figure, orientation, and motion) and then a simulator will be established. During simulation, a basic functional conceptual system can be constructed, representing categorization, propositions, abstract concepts, and their kind. Therefore, we assume that the perceptual symbols that involve the perceptive motion in music and words can be activated just because our corresponding sensorimotor experience comes into play in understanding the motion concept implicated in words. On this account, action verbs and animate nouns can more easily trigger our sensorimotor experience in the brain so as to make the processing at ease in the motion relatedness task. Music, a motion-based art that unfolds over time, surely contains certain motion information. Hence, we may infer that action verbs and animate nouns (relative to state verbs and inanimate nouns) are easier to process in the music priming condition just on account of the more shared motion information (revealed by the semantic index N400), for sensory-motor cortex may have already been activated as soon as the music is heard by participants. Our results, along with other related studies (Stanfield and Zwaan, 2001; Zwaan et al., 2002; Wang and Zhao, 2020), support the perceptual symbol theory that perceptual symbols of referent are activated during language comprehension (Barsalou, 1999).

### Motion Concept Modulated by Embodiment

In our experiments, the motion concept is taken as the referencing point to examine how different music may exert impacts on verbs and nouns. The experimental design in this way is based on the belief that music and words are encoded with the shared motion concept (though in a different manner), a high level of meaning across modalities. The results justified the belief, as indicated by the N400 effect. We argue that the different processing patterns associated with the four classes of words and music are attributed to the diverse degree of embodiment involved in the two domains (music and words).

As demonstrated above, the music with tempo changes is connected with more musical motion, and the music without tempo change with less musical motion whereas action verbs and animate nouns are judged with more motion information than state verbs and inanimate nouns. As a consequence, a decreased N400 was observed in action verbs and animate nouns under the priming condition of music with tempo changes, but verbs showed reversed pattern and nouns showed no obvious N400 difference under the priming condition of music without tempo changes. In our everyday life, physical motion is easily and frequently embodied and encoded as a motion concept in both music and words metaphorically. In association with our results, it appears safe to infer that the more embodied motion is involved in words, the smaller the evoked N400 amplitudes are. Such an inference is favored by few studies indirectly, e.g., the motion concept in music will create an expectation of the target pictures even though it is irrelevant to the task (Hedger et al., 2013; Zhou et al., 2015). The motion concept as a high-level meaning exists across domains, indexed by the N400 effect electrophysiologically, which is in accordance with Zhou et al.'s (2015) study. Just like the case in pictures, the motion concept of verbs and nouns can be well-comprehended metaphorically by virtue of individuals' sensorimotor experience.

In light of embodiment theories, semantic processing is wholly reliant on sensorimotor experience (Lakoff and Johnson, 1999, p. 151; Glenberg and Kaschak, 2002), our bodily experience non-technically. Likewise, the comprehension of the motion concept in words also cannot be separated from embodied experience. Given that our participants performed a motion relatedness task, processing words in our studies was not decoding the literal meaning but interpreting the motion clues of words. Our results are consistent with former studies in which perceptive information (such as orientation, motion features, location, and shape of objects) encoded by language was understood by embodied experience (e.g., Stanfield and Zwaan, 2001; Zwaan et al., 2002; Wang and Zhao, 2020).

Overall, our results indicate that the motion concept is shared by music and words on the embodiment basis and this sharing is indexed by the N400 component, adding further evidence to the view that music and language are supposed to share the neural mechanism of meaning processing (Koelsch et al., 2004; Koelsch, 2011; Jiang, 2016).

## Conclusion

Music and language signal two domains human beings use to exchange intentions and meanings despite their key differences (specificity, compositionality, and communicativeness) (Slevc and Patel, 2011). The current study demonstrates that music and language are conceptually or semantically correlated with the general motion concept *via* embodiment. Our results obtained indicate that the processing of verbs and nouns is influenced by music tempo types and N400 indexes the motion concept shared by music and words. On the one hand, action verbs and animate nouns are easier to process than state verbs and inanimate nouns, respectively, due to the more motion information they own in the motion-related tasks. On the other hand, four types of words can be distinguished perceptually when primed by different music tempos. Moreover, these findings demonstrate that the motion concept exists regardless of the modalities and domains the priming stimuli and target stimuli are involved in, favoring the embodiment theory in the comprehension of musical and linguistic meaning.

In conclusion, our experiments extend the previous studies by showing that music tempos can conceptually prime words in the language and adds evidence to the hypothesis that music and language share the neural mechanism of meaning processing. The challenge for future research is to explore the neural mechanism of other aspects of meaning assumed to be shared by music and language.

## Data Availability Statement

The raw data supporting the conclusions of this article will be made available by the authors, without undue reservation.

## Ethics Statement

The studies involving human participants were reviewed and approved by Biomedical Ethics Committee of Qufu Normal University. The patients/participants provided their written informed consent to participate in this study.

## Author Contributions

TZ, YL, and HL conceived the study. YL and TW performed the experiments. YL collated and analyzed the data. YL drafted the first manuscript, which TZ and SZ revised. All authors edited the final version of the manuscript and have approved it for publication.

## Funding

This work was supported by the Fundamental Research Funds for the Central Universities under the No. 2242022R10100.

## Conflict of Interest

The authors declare that the research was conducted in the absence of any commercial or financial relationships that could be construed as a potential conflict of interest.

## Publisher's Note

All claims expressed in this article are solely those of the authors and do not necessarily represent those of their affiliated organizations, or those of the publisher, the editors and the reviewers. Any product that may be evaluated in this article, or claim that may be made by its manufacturer, is not guaranteed or endorsed by the publisher.

## Footnotes

^1^In Koelsch (2011), extral-musical meaning is defined as the interpretation of the musical information associated with external world, which means musical patterns or acoustic properties resembles the emotion state, sounds of objects, qualities of entity, abstract concept and their like. For example, a music excerpt may sound like “a bird”; the accelerating music tempos may sound like “running” movement.
